# Effects of emotional labor and sleep quality on depression in older apartment security workers in Korea: a cross-sectional study

**DOI:** 10.7586/jkbns.25.040

**Published:** 2025-08-26

**Authors:** Seungmi Park, Sujin Kang, Hye Ok Park, Youngji Kim

**Affiliations:** 1 College of Nursing & Research Institute of Nursing Science, Chungbuk National University, Cheongju, Korea; 2 College of Nursing, The Catholic University of Korea, Seoul, Korea; 3 Kumudini Nursing College, Mirzapur, Tangail, Bangladesh; 4 Department of Nursing, College of Nursing and Health, Kongju National University, Gongju, Korea

**Keywords:** Aged, Emotional exhaustion, Sleep quality, Depression

## Abstract

**Purpose:**

Korea’s population is aging rapidly, and many older adults are now working as apartment security workers after retirement. They often experience poor working conditions and emotional labor, which can lead to depression. Although factors such as age, health, job insecurity, emotional labor, and sleep problems relate to depression in older workers, little evidence exists regarding older apartment security workers. This study, therefore, examined the effects of emotional labor and sleep quality on depression in these individuals.

**Methods:**

Between August and December 2023, 209 older apartment security workers participated in the study, completing a questionnaire that assessed their emotional labor, sleep quality, and depression. Data were examined using descriptive statistics, the independent t-test, Mann-Whitney U test, analysis of variance, and multiple regression analysis.

**Results:**

This study found that 206 participants (98.6%) experienced poor sleep quality, and 70 participants (33.5%) reported feeling depressed. The multiple linear regression analysis revealed that family type (β = .30), sleep quality (β = .29), emotional labor (β = .16), and unfair dismissal (β = .14) were significant predictors of depression, with an explanatory power of 25.0% (F = 18.26, *p* < .001).

**Conclusion:**

Depression and inadequate sleep were found to be highly prevalent in older security workers. The findings suggest that social isolation, inadequate sleep, increased emotional labor, and unstable employment may increase the risk of depression. Therefore, routine screening for these predictors may be beneficial, and comprehensive nursing interventions should be developed to reduce depression among older apartment security workers.

## INTRODUCTION

Globally, all regions and places experience population ageing. Of all, Korea’s population is aging very rapidly. The working-age population and the elderly dependent population are decreasing, threatening the lives of the elderly [1]. As a result, people in their 60s are excluded from complete retirement and are opting to maintain their careers through measures like extending their retirement age or choosing reemployment after retirement [2]. In this situation, security worker is one of the most preferred occupations for seniors facing difficulties in employment and job search due to their age [3]. Currently, 21.3% of elderly workers aged 65 and above are working as security workers in Korea [4].

Apartment security workers are classified as "Elementary Workers" under the "Cleaning and Guard Related Elementary Occupations" category of the Korean Standard Classification of Occupations, which includes facility security [4]. Their primary responsibilities include monitoring activity both inside and outside the apartment building, regulating guest access, overseeing parking, accepting and documenting mail deliveries, maintaining cleanliness, sorting recyclables, and dealing with tenants’ concerns [4]. Elderly apartment security workers work in bad working circumstances, engaged in 24-hour and night shifts, and sleeping in small security guard posts [4,5]. Many demographic and health factors, including age, current smoking status, alcohol consumption [6], and perceived health status [7], have been linked to depression in security workers. Other work-related factors include job insecurity, low pay, threats and harassment from superiors, experience of violence [6], irregular work schedules [8], short rest time [9], and emotional labor [10]. Notably, the sleep quality of shift workers, including apartment security workers, has been reported to be closely associated with depression [11].

Emotional labor is the process by which employees regulate or manage their feelings and expressions to achieve organizational goals [12]. When dealing with complaints and interacting with tenants, apartment security workers engage in emotional labor [13,14]. The majority of them are indirectly employed through subcontractors or service businesses, and because they are required to renew contracts annually, they are forced to endure unjust treatment by residents in order to minimize conflicts. Such emotional labor impacts them in various ways and leads to increased job stress and decreased job satisfaction, and it can result in cardio-cerebrovascular diseases, as well as various mental health issues including anxiety, agitation, anger disorders, social phobia, panic disorders, depression, and suicide [15]. Apartment security workers who repeatedly work shifts and night shifts experience low sleep quality. Apartment security workers had lower sleep quality compared to other shift workers in different occupations (nurses, male manufacturing workers) or elderly residents in the community [13]. Above all, shift work is related with impacts on sleep, depressed mood, and even suicidal ideation [16].

Therefore, in this study, the low sleep quality and emotional labor of apartment security workers, who repeatedly work shifts, were considered important factors that could directly affect depression and were included as key independent variables.

Concerns about the work environment and mental health of apartment security workers have grown in society as a result of reports of verbal and physical abuse, as well as some incidents of burnout [17]. According to studies, approximately 21~30% of apartment security workers have experienced depression with an average of around four depressive symptoms reported [6]. In comparison, the prevalence of depressive symptoms among community-dwelling old people was approximately 11.3% according to 2023 data from the Ministry of Health and Welfare, and it was reported to be about 13.5% in 2020 [18]. The markedly higher rate among elderly apartment security workers suggests a significantly elevated risk in this demographic. Depression can occur across all age groups, but it is particularly common in old age, being one of the most prevalent mental disorders among the elderly [19]. In addition to negatively affecting an employee's physical and mental well-being, depression also was associated with lower productivity (i.e., absenteeism and presenteeism) [20].

Although awareness of emotional labor, sleep quality and depression have increased among health professionals, the relationship between emotional labor, sleep quality, and depression has not been thoroughly studied in our participants. A lack of research in this area highlights an insufficient understanding of mental health concerns among apartment security workers.

The aim of current study was to determine the level of emotional labor, sleep quality, and depression and to identify the risk factors on depression among elderly apartment security workers.

## METHODS

### 1. Study design

This cross-sectional descriptive study was conducted to examine the effects of emotional labor and sleep quality on depression among elderly apartment security workers.

### 2. Participants

This study targeted males aged 65 and above working as security workers in residential buildings in 10 regions nationwide, four metropolitan cities (Incheon, Daejeon, Gwangju, and Daegu), and six provinces (Gyeonggi, Gangwon, Chungbuk, Chungnam, Jeonbuk, and Gyeongbuk). Security workers working in offices, commercial facilities, and public facilities were excluded from the study. Convenience sampling was employed among participants who understood the purpose of the research and voluntarily consented to participate. The sample size was estimated using G*Power 3.1.9.7 [21], with the alpha level set at .05, the power set at 0.8, the effect size set at 0.15 based on a previous study on apartment security workers [13], with 28 predictors. The minimum sample size was estimated at 181. Taking a dropout rate of 20% into account, 227 participants were surveyed, with a total of 209 participants included in the final analysis after excluding 18 respondents who provided unreliable responses.

### 3. Instruments

#### 1) Participants characteristics

The general questionnaire included demographic characteristics (age, education, marital status, family type, religion, perceived economic status, average monthly income), health characteristics (alcohol consumption, current smoking, weekly caffeine intake, presence of chronic diseases, physical activity, perceived health status), and work-related characteristics (working area, number of apartment buildings, management entity, total duration of service as apartment security workers, weekly working hours, shift system, separate break room, rest time guaranteed, previous experience of unfair dismissal, anxiety about unfair dismissal, experience of verbal/physical violence, physically demanding tasks).

Average monthly income was categorized based on 1,975,000 Korean Won (KRW), the 2023 minimum income for temporary workers. Caffeine intake was assessed by investigating the consumption frequency (number of cups) of beverages containing caffeine (coffee, green tea/black tea, soft drink, or energy drink) over the past week. The weekly caffeine intake per person was calculated based on the caffeine content provided for each beverage. Physical activity was assessed using the International Physical Activity Questionnaire (IPAQ). We surveyed the duration and frequency of vigorous-intensity physical activity, moderate-intensity physical activity, walking, and sedentary time for at least 10 minutes per day over the past week by using IPAQ. This physical activity was calculated into Metabolic Equivalent Task (MET) scores according to IPAQ guidelines. The participants with a weekly physical activity level of less than 600 METs were classified as the inactive group, while those with 600 METs or more were classified as the active group [22].

#### 2) Emotional labor

Emotional labor of participants was measured using the "Korean Emotional Labor Scale (K-ELS®11)" developed by Jang et al. [14], composed of 11 items. K-ELS®11 consists of four sub-domains (‘emotional regulation’ with two items, ‘emotional dissonance’ with three items, ‘organizational monitoring’ with two items, ‘organizational protective system for emotional labor’ with four items), and a total of 11 items. It is scored on a 4-point Likert scale with a total score of 44, where higher scores indicate a higher degree of emotional labor. The Cronbach’s alpha of the K-ELS®11 was .79 at the time of its development [14], and .81 in the present study.

#### 3) Sleep quality

The sleep quality of participants refers to their everyday sleep habits experienced over the past 4 weeks. The sleep quality of elderly apartment security workers was measured using the Korean version of the Pittsburgh Sleep Quality Index (PSQI-K), adapted by Sohn et al. [23] from Buysse et al.'s [24]. The PSQI-K consists of seven sub-domains (sleep quality, sleep latency, sleep duration, habitual sleep efficiency, sleep disturbance, use of sleep medication, daytime dysfunction) and a total of 19 items. Each item is scored on a scale of 0 to 3 points, with the total score ranging from 0 to 21 points. The higher the score is, the poorer one’s sleep quality is, and individuals with a score of 6 or higher are classified as poor sleepers. The Cronbach’s alpha of the PSQI-K at the time of its development was 0.84 [23], while it was .45 in the present study. Since the PSQI consists of seven subcomponents (e.g., subjective sleep quality, sleep latency, sleep duration, etc.), it has a multidimensional structure, which may result in low internal consistency and, consequently, a low Cronbach’s alpha [25].

#### 4) Depression

The depression of participants refers to their mood over the past week. The short form (15 items) of the Geriatric Depression Scale, developed by Yesavage and Sheikh [26], which was adapted into the Korean version by Kee [27] as the Geriatric Depression Scale Short Form-Korea (GDSSF-K), was used to assess their depression. It consists of a total of 15 items, and the participants were to answer them with either 'yes' or 'no.' Negative items were reverse-scored, resulting in scores ranging from 0 to 15. The cut-off point for depression diagnosis is 5 points, with scores above 5 indicating a higher tendency toward depression. The Cronbach’s alpha was .81 in this study. The Cronbach’s alpha of the GDSSF-K at the time of its development was .79 [27], while it was .81 in the present study.

### 4. Data collection

The data for this study were collected from August 1 to December 15, 2023. The research assistants trained in data collection for this study visited the apartment and collected data. Participants who heard an explanation of the purpose of this study from the researcher and voluntarily agreed to participate completed a written consent form and then participated in the survey using a self-administered questionnaire. It took approximately 15 to 20 minutes to complete the survey, and participants were compensated with a gift for their participation. As all of the participants were elderly, research assistants read the questions aloud while conducting the survey if they experienced difficulty in reading the items in the survey. To provide consistent explanations about the survey process, the researchers held two meetings in advance to standardize the data collection procedures.

### 5. Data analysis

Descriptive analysis of sociodemographic data and study variables was undertaken. The dependent variable in the present study was depression. An independent t test and a one-way analysis of variance (post-analysis verification by Scheffé) were used to investigate the differences in depression scores among the various groups in terms of demographic and health- and work-related characteristics. Pearson correlation was used for bivariate analysis to identify significant associations among the continuous variables. Statistically significant factors from the abovementioned inferential statistics were then entered into a regression analysis. Finally, a multiple linear regression analysis was conducted to determine the factors that were associated with depression at a level of significance of .05 or higher. All data were analyzed using IBM SPSS version 23.0 (IBM Corp., Armonk, NY, USA).

Multiple regression analysis was conducted to identify the factors influencing depression among the participants. Variables that showed significant associations with depression in the univariate analysis, including marital status, family type, weekly caffeine intake, perceived health status, working area, number of apartment buildings, management entity, previous experience of unfair dismissal, anxiety about unfair dismissal, experience of verbal/physical violence, physically demanding tasks, emotional labor, and sleep quality, were entered into the analysis using the stepwise method. Multiple regression analysis was conducted using the stepwise method to identify the most significant predictors of depression while minimizing multicollinearity among the variables [28]. This method was considered appropriate because the study included a relatively large number of potential predictors, and the stepwise approach helps build a parsimonious model by automatically selecting variables with significant explanatory power. Categorical variables such as marital status, family type, weekly caffeine intake, perceived health status, working area, number of apartment buildings, management entity, previous experience of unfair dismissal, anxiety about unfair dismissal, experience of verbal/physical violence, and physically demanding tasks were treated as dummy variables for analysis.

### 6. Ethical considerations

This study was approved by the Institutional Review Board of Kongju National University (approval number: KNU_IRB_2023-021). Prior to participation in the survey, the purpose and the study methods were explained to the participants, and to ensure confidentiality and anonymity, the participants were informed that the collected data would be used solely for research purposes. Additionally, they were notified that participation in the survey was voluntary, and participants could withdraw at any time without any repercussions. Only those who fully understood and voluntarily agreed to the aforementioned conditions were allowed to participate in the survey. Written informed consent forms were gathered from the participants before data collection.

## RESULTS

### 1. Levels of emotional labor, sleep quality, and depression

209 men participants were included in this analysis. Descriptive statistics for study variables are presented in Table 1. The average total score for emotional labor was 24.90 (± 4.75), and the average total score for sleep quality was 9.63 (± 2.16), with 206 participants (98.6%) classified as poor sleepers. The average total score for depression was 3.88 (± 3.31), and 70 participants (33.5%) scored 5 or higher, indicating depression.

### 2. Participant characteristics

The background data (demographic and health characteristics) of the participants are presented in Table 2. The mean age of these participants was 69.28 (± 3.66) years, with 189 participants (90.4%) being aged 65~69 years. A total of 126 participants (60.3%) were educated to the high school or higher. 174 participants (83.3%) were married, and 179 participants (85.6%) lived with family. A total of 116 participants (55.5%) had a religion. 183 participants (87.6%) were classified as poor by perceived economic status, and the average monthly income was 2,144,300 KRW (± 346,100), with 182 participants (87.1%) earning 1,975,000 KRW or more.

### 3. Differences in depression based on demographic and health characteristics of the participants

The following are the data regarding the demographic characteristics of the participants. Depression showed statistically significant differences only in marital status (t = −2.23, *p* = .029) and whether living with family (t = 4.09, *p* < .001) in terms of demographic characteristics.

The following are the data regarding the health characteristics of the participants. 129 participants (61.7%) consumed alcohol, 143 participants (68.4%) were non-smokers, 157 participants (75.1%) had a weekly caffeine intake of more than 400 mg, and 149 participants (71.3%) had chronic diseases. A total of 165 participants (78.9%) had a physical activity level of 600 MET or more, and 123 participants (58.9%) reported poor perceived health status. Depression showed statistically significant differences only in weekly caffeine intake (t = −2.16, *p* = .033) and perceived health status (t = 4.24, *p* < .001) in terms of health characteristics (Table 2).

### 4. Differences in depression based on work-related characteristics of the participants

The following are the data regarding the work-related characteristics of the participants. 171 participants (81.8%) worked in provincial areas, 142 participants (67.9%) worked in apartments with more than five buildings, and 179 participants (85.6%) were affiliated with a contract management. The average of total duration of service as apartment security workers was 6.38 (± 4.55) years, with 113 participants (54.1%) having worked for more than 5 years. The average of weekly working hours were 72.61 (± 18.38) hours, with 113 participants (54.1%) working 72 hours or more per week. Most participants, 203 individuals (97.1%), worked 24-hour alternating shifts. One hundred thirty-eight participants (66.0%) did not have a separate break room. When it comes to rest time guaranteed, 99 participants (47.4%) could not leave their post during rest times and had to handle urgent matters if they arose. One hundred eighty participants (86.1%) had no previous experience of unfair dismissal, but 145 participants (69.4%) had anxiety about unfair dismissal. One hundred fourteen participants (54.5%) had experienced verbal or physical violence, and 146 participants (69.9%) performed physically demanding tasks (Table 3).

The participants' depression levels showed statistically significant differences based on several work-related characteristics: working area (t = 2.34, *p* = .020), number of apartment buildings (t = −2.53, *p* = .012), management entity (t = −4.12, *p* < .001), previous experience of unfair dismissal (t = −2.49, *p* = .014), anxiety about unfair dismissal (t = −4.89, *p* = .001), experience of verbal/physical violence (t = −3.32, *p* = .001), and physically demanding tasks (t = −3.02, *p* = .003) (Table 3).

### 5. Correlations among emotional labor, sleep quality, and depression

The participants' depression showed a statistically significant positive correlation with both emotional labor (r = .27, *p* < .001) and sleep quality (r = .33, *p* < .001). Emotional labor also showed a significant positive correlation with sleep quality (r = .21, *p* = .002) (Table 4).

### 6. Factors related to depression

Prior to conducting the stepwise multiple regression analysis, the assumptions of multiple linear regression were tested. The skewness and kurtosis values for the main variables were all within an absolute value of 2, indicating that the normality assumption was satisfied. The residual scatterplot showed no distinct pattern, confirming that the assumption of homoscedasticity was met. The Durbin–Watson statistic was 1.895, which is close to 2, indicating that there was no autocorrelation and that the residuals were independent. Tolerance values ranged from .93 to .98, and variance inflation factor values ranged from 1.02 to 1.08, which are well below the threshold of 10, indicating that multicollinearity was not an issue. The condition index ranged from 1.00 to 14.52, which is below the commonly accepted threshold of 15, further supporting the absence of multicollinearity.

The factors contributing to depression is shown in Table 5. The regression model for depression was significant (F = 18.26, *p* < .001). Living alone, poorer sleep, higher emotional labor, and unfair dismissal accounted for 25% of the total variance in depression. Living alone (β = .30, *p* < .001), having poorer sleep (β = 0.29, *p* < .001), having higher emotional labor (β = .16, *p* = .012), and having experienced an unfair dismissal in the past (β = .14, *p* = .023) were all found to be significantly associated with depression.

## DISCUSSION

This study was conducted to investigate the effects of emotional labor and sleep quality on depression among elderly apartment security workers in Korea. The results confirmed that living alone, poorer sleep quality, higher levels of emotional labor, and previous experience of unfair dismissal were all significantly associated with an increased risk of depression in this group. Notably, 33.5% of elderly apartment security workers experienced depression (cutoff point ≥ 5), which is somewhat higher than the reported range of 21~30% in previous studies [6] and markedly higher than the 11.3% prevalence among community-dwelling older adults in Korea [18]. This study also shows that elderly apartment security workers face multiple factors that may contribute to depression, including advanced age, chronic health conditions, and work-related risks such as 24-hour rotational shifts, unstable employment, and long working hours that exceed the legal limit of 52 hours per week (average weekly working hours of 72.61) [16]. These findings highlight that living alone, poor sleep quality, emotional labor, and experiences of unfair dismissal play important roles in increasing depression risk, underscoring the need for comprehensive interventions that address these factors together. Therefore, targeted measures to manage emotional labor, improve sleep quality, support employment stability, and reduce social isolation are essential to alleviate depression in this vulnerable group.

First, living alone is the factor that had the greatest impact on depression among elderly apartment security workers. According to the 2023 Elderly Survey, 11.3% of the overall elderly population experienced depression, with symptoms worsening with age [18]. Furthermore, the depression rate among elderly individuals without a spouse was 8.3% higher than those with a spouse [18]. A study by Srivastava et al. [29] also found that elderly individuals living alone had a 16% higher likelihood of experiencing depression compared to those living with family. Specifically, elderly individuals who experienced bereavement and lived alone had a 56% higher likelihood of experiencing depression.

Elderly individuals living alone are more likely to experience depression due to loneliness and lack of social support. Those who have experienced bereavement and live alone suffer from depression even more as they face stress from spousal loss and deficiency in emotional support [29]. When elderly individuals living alone experience higher levels of depression, it can lead to higher rates of illness due to irregular meals and inadequate nutritional intake, resulting in deteriorating health [30]. Despite these serious circumstances, there is a lack of understanding and awareness among the elderly and local communities regarding mental illnesses in the elderly, including depression, leading to inadequate diagnosis and treatment [31]. Therefore, multifaceted efforts are needed to raise awareness of depression and strengthen social support for this group. For example, a recent systematic review and meta-analysis demonstrated that community-based support group interventions significantly reduced depression levels among elderly individuals living alone by enhancing their social connections and perceived emotional support [32]. To this end, it is necessary not only to link with community mental health resources but also to conduct regular depression screenings and early interventions, and to operate community-based support group programs for elderly individuals living alone to help reduce their emotional isolation.

Secondly, poor sleep quality is the second most influential factor affecting depression among elderly apartment security workers. It was found that 98.6% of elderly apartment security workers were classified as poor sleepers, indicating that the majority of participants have poor sleep quality. This proportion is higher than the poor sleep quality rate (93.4%) of apartment security workers reported by Kim et al. [13]. According to Han’s study [33], among workers aged 60 and above, those who were classified as ‘Elementary workers’ (including apartment security workers) showed the highest prevalence of sleep disorders at 4.2%, with 36.6% experiencing depression accompanied by sleep disorders. Sleep and depression are closely related, as changes in the quality and quantity of sleep can lead to changes in neural circuits and neurotransmitters associated with depression, either exacerbating or alleviating depression [34]. The quality of sleep is known to be an essential factor in assessing the risk of depression among shift and night workers [35,36]. In other words, poor sleep quality is considered one of the main causes of mental health issues, including depression, among shift and night workers [16]. Fatigue resulting from long working hours is believed to be the primary cause of poor sleep quality of apartment security workers [13].

However, the risk of depression due to long working hours can be reduced, even for shift workers, if they are guaranteed sufficient time for a rest [9,37]. Therefore, to improve the sleep quality of apartment security workers who work long hours in cramped security guard post while balancing rest and sleep, it is essential to secure guaranteed rest time and provide separate break rooms for rest and sleep [4,13]. To be more specific, it is necessary to take naps of less than 30 minutes during rest times to reduce fatigue and make themselves feel better, ensure at least 7 hours of sleep time for fatigue recovery on days off, refrain from caffeine intake and alcohol consumption, and encourage adequate nutrient intake to improve sleep quality [13]. It is particularly important to ensure that residents do not disrupt the designated rest and sleep times of apartment security workers by attaching visible notices to warn the residents. The management office should also adjust nighttime duties to guarantee their time to rest and sleep effectively [4]. It is also necessary to create an atmosphere that enables the provision of separate break rooms through cooperation between local governments, management offices, and residents [5].

Thirdly, emotional labor is the third most influential factor affecting depression among elderly apartment security workers. Apartment security workers are inevitably involved in emotional labor, catering to various demands of residents while suppressing and restraining their own emotions. Specifically, unpleasant comments and conduct from tenants can increase job stress and cause emotional fatigue in apartment security workers [4]. Such job stress ultimately has a negative impact on the level of depression among apartment security workers. In other words, chronic stress due to emotional labor increases the levels of inflammatory cytokines, continuously stimulating the hypothalamic-pituitary-adrenal axis, leading to anxiety and depression [38,39]. Therefore, it is crucial to adhere to the "Anti-bullying law for security workers" and clearly define the permissible scope of duties for apartment security workers to reduce the negative impact of emotional labor when they interact with the residents. Furthermore, residents who are prone to complaints or disagreements should have standardized response manuals established for them, and professional counseling help should be made available in the event of emotional labor situations [4,13]. Above all, there needs to be an improvement in residents' awareness and attitude toward the value and importance of the duties of apartment security workers [13]. In addition, nursing intervention programs for mental health that include training on self-expression techniques, work-life balance, self-encouragement, emotion regulation, anger regulation training, communication, stress-relieving skills, etc. should be prepared for apartment security workers [4].

Lastly, experience of unfair dismissal is the fourth most influential factor affecting depression among elderly apartment security workers. In this study, 13.9% of elderly apartment security workers were found to have experienced unfair dismissal, which is a higher figure than the national survey results of apartment security workers in 2019 (11.1%) [5]. Unfair dismissal refers to termination during the employment contract period and termination without justifiable reasons upon expiration of the employment contract [5]. Employees who have experienced dismissal may suffer from physical symptoms such as headaches, backache, skin problems, muscular pain and eye strain. [40].

Most apartment security workers are indirect temporary workers who sign contracts with outsourcing management companies through service contracts, and they often experience job insecurity at each renewal of the service contract due to the prevalence of short-term contracts [5]. The Act on Prohibition of Age Discrimination in Employment and Elderly Employment Promotion (Article 4-4) prohibits discrimination against workers based on age in retirement and dismissal without rational reasons [41]. However, demands for retirement or unfair dismissal based on age are frequent among apartment security workers aged 60 and above. The problem is that in most cases, there is a lack of awareness about these acts of age discrimination [41]. To address these issues, it is necessary to extend the retirement age of apartment security workers (setting the recommended retirement age to 70) and to secure stability in employment for older workers by employing them in temporary contract positions based on their health conditions even after retirement [5]. Additionally, local governments should make efforts to improve the employment environment of apartment security workers by enacting ordinances to improve their treatment, selecting exemplary sites for employment stability annually, providing incentives such as support for libraries, and conducting diverse efforts such as education on labor rights and monitoring targeting representatives of residents and management office personnel [5].

Therefore, it is necessary to implement regular educational programs aimed at improving awareness of depression among elderly apartment security workers, thereby enhancing their accurate understanding and knowledge of depression. In other words, it is important to collaborate with local mental health centers and educate security workers through specialized psychiatric nurses to raise awareness of depression and suicide risk, screen and diagnose depression early, and provide education on seeking counseling and treatment from professionals if necessary. Nursing interventions emphasizing social connections are particularly necessary for elderly apartment security workers who are living alone, and these interventions can be implemented by encouraging frequent communication with family, friends, and close acquaintances, participating in support groups, and promoting individual efforts such as regular exercise, volunteering, hobbies, maintaining a balanced diet, and healthy eating habits [42].

While this study attempted to avoid bias by collecting data from 10 regions across the country using convenience sampling, the conclusions are limited in their applicability to the total population of apartment security workers. Since this study focused only on emotional labor, sleep quality, and depression, further research is necessary to explore various physical and mental health factors as well as labor environment factors that may influence depression among elderly apartment security workers. Nevertheless, this study suggests policy implications such as ensuring rest time and separate break room, stabilizing employment for older workers, and providing education on labor rights to alleviate depression among apartment security workers. Additionally, the significance of this study lies in highlighting the need for developing and implementing nursing intervention programs for promoting mental health aimed at mitigating emotional labor, improving awareness of depression, and early diagnosis and treatment.

In conclusion, these findings highlight the urgent need for multi-level interventions to reduce depression among elderly apartment security workers by addressing living conditions, work-related stressors, and mental health support. Future research should continue to expand on these factors and develop evidence-based nursing interventions and policy measures.

## CONCLUSION

In summary, depression and inadequate sleep are highly prevalent in elderly security workers. The findings suggest that social isolation, inadequate sleep, increased emotional labor and unstable employment may increase the risk of depression. Therefore, routine screening for the predictors of those workers may also be beneficial. Also, comprehensive nursing interventions should be developed to reduce depression among elderly apartment security workers, with a particular focus on raising awareness of depression, maintaining social support networks, improving sleep quality through effective use of rest times, and relieving emotional labor through stress management strategies.

## Figures and Tables

**Table 1.** Descriptive Statistics of Emotional Labor, Sleep Quality, and Depression (N = 209)

Variables	n (%)	M ± SD	Min ~ Max
Emotional labor		24.90 ± 4.75	11 ~ 34
Sleep quality		9.63 ± 2.16	5 ~ 16
Good sleeper (0~5)	3 (1.4)		
Poor sleeper (≥ 6)	206 (98.6)		
Depression		3.88 ± 3.31	0 ~ 13
Normal (< 5)	139 (66.5)		
Depressed (≥ 5)	70 (33.5)		

M = Mean; SD = Standard deviation; Min = Minimum; Max = Maximum.

**Table 2.** Depression by Demographic and Health Characteristics (N = 209)

Variables	n (%) or M ± SD	Depression	
		M ± SD	t/F/Z (*p*)
Demographic characteristics			
Age (year)	69.28 ± 3.66		
65 ~ < 70	189 (90.4)	106.18^†^	−0.87^‡^ (.383)
≥ 70	20 (9.6)	93.88^†^	
Education			
< High school	83 (39.7)	4.1 ± 3.48	0.78 (.435)
≥ High school	126 (60.3)	3.73 ± 3.19	
Marital status			
Married	174 (83.3)	3.62 ± 3.14	−2.23 (.029)
Others	35 (16.7)	5.17 ± 3.84	
Family type			
Living alone	30 (14.4)	6.63 ± 4.14	4.09 (< .001)
Living with family	179 (85.6)	3.41 ± 2.91	
Religion			
No	93 (44.5)	3.87 ± 3.21	−0.02 (.986)
Yes	116 (55.5)	3.88 ± 3.40	
Perceived economic status			
Bad	183 (87.6)	106.76^†^	−1.13^‡^ (.260)
Good	26 (12.4)	92.60^†^	
Average monthly income (10,000 KRW)	214.43 ± 34.61		
< 197.5	27 (12.9)	114.48^†^	−0.88^‡^ (.379)
≥ 197.5	182 (87.1)	103.59^†^	
Health characteristics			
Alcohol consumption			
No	80 (38.3)	3.85 ± 3.58	−0.09 (.930)
Yes	129 (61.7)	3.89 ± 3.14	
Current smoking			
No	143 (68.4)	3.64 ± 3.23	−1.50 (.135)
Yes	66 (31.6)	4.38 ± 3.44	
Weekly caffeine intake			
< 400 mg	52 (24.9)	3.11 ± 2.73	−2.16 (.033)
≥ 400 mg	157 (75.1)	4.13 ± 3.45	
Presence of chronic diseases			
No	60 (28.7)	3.23 ± 3.07	−1.79 (.075)
Yes	149 (71.3)	4.13 ± 3.37	
Physical activity			
Inactive (< 600 MET)	44 (21.1)	4.41 ± 3.20	1.29 (.229)
Active (≥ 600 MET)	165 (78.9)	3.73 ± 3.33	
Perceived health status			
Bad	123 (58.9)	4.62 ± 3.54	4.24 (< .001)
Good	86 (41.1)	2.81 ± 2.60	

M = Mean; SD = Standard deviation; KRW = Korean won; MET = Metabolic equivalent of task.

^†^Mean ranking; ^‡^Mann-Whitney U test.

**Table 3.** Depression According to Work-related Characteristics (N = 209)

Variables	n (%) or M ± SD	Depression	
		M ± SD	t/F/Z (*p*)
Working area			
Metropolitan areas	38 (18.2)	5.00 ± 3.47	2.34 (.020)
Others^§^	171 (81.8)	3.63 ± 3.22	
Number of apartment buildings			
≤ 5	67 (32.1)	3.04 ± 3.05	−2.53 (.012)
> 5	142 (67.9)	4.27 ± 3.36	
Management entity			
Autonomous management	30 (14.4)	2.20 ± 2.20	−4.12 (< .001)
Management entrusted	179 (85.6)	4.16 ± 3.38	
Total duration of service as apartment security workers (year)	6.38 ± 4.55		
< 5	96 (45.9)	3.91 ± 3.42	0.14 (.889)
≥ 5	113 (54.1)	3.85 ± 3.21	
Weekly working hours	72.61 ± 18.38		
≤ 72	96 (45.9)	3.59 ± .37	1.22 (.225)
> 72	113 (54.1)	3.04 ± .29	
Shift system			
24-hour shifts	203 (97.1)	104.51^†^	−0.68^‡^ (.497)
Others^∥^	6 (2.9)	121.42^†^	
Separate break room			
No	138 (66.0)	3.91 ± 3.32	0.23 (.820)
Yes	71 (34.0)	3.80 ± 3.30	
Rest time guaranteed			
Can leave workplace & use rest time freely	51 (24.4)	3.37 ± 2.81	0.97 (.382)
Can’t leave workplace, but possible to freely nap, etc.	59 (28.2)	3.83 ± 3.36	
Can’t leave workplace & need to deal with urgent matters	99 (47.4)	3.88 ± 3.31	
Previous experience of unfair dismissal			
No	180 (86.1)	3.65 ± 3.22	−2.49 (.014)
Yes	29 (13.9)	5.28 ± 3.55	
Anxiety about unfair dismissal			
No	64 (30.6)	2.56 ± 1.97	−4.89 (.001)
Yes	145 (69.4)	4.46 ± 3.60	
Experience of verbal/physical violence			
No	95 (45.5)	3.08 ± 2.96	−3.32 (.001)
Yes	114 (54.5)	4.54 ± 3.39	
Physically demanding tasks			
No	63 (30.1)	2.94 ± 2.70	−3.02 (.003)
Yes	146 (69.9)	4.28 ± 3.46	

M = Mean; SD = Standard deviation.

^†^Mean ranking; ^‡^Mann-Whitney U test; ^§^Six provinces (Gyeonggi, Gangwon, Chungbuk, Chungnam, Jeonbuk, Gyeongbuk); ^∥^12-hour shifts, only daytime work, etc.

**Table 4.** Correlations of Depression, Emotional Labor, and Sleep Quality (N = 209)

Variables	Depression	Emotional labor r (*p*)	Sleep quality
	r (*p*)		
Emotional labor	.27 (< .001)	1	-
Sleep quality	.33 (< .001)	.21 (.002)	1

**Table 5.** Factors Affecting Depression in Participants (N = 209)

Variables	B	SE	β	t	*p*
(Constant)	−3.68	1.26		−2.91	.004
Family type^†^ (Living alone)	2.77	0.57	0.30	4.86	< .001
Sleep quality	0.44	0.09	0.29	4.67	< .001
Emotional labor	0.11	0.04	0.16	2.52	.012
Previous experience of unfair dismissal^‡^ (Yes)	1.33	0.58	0.14	2.30	.023
F = 18.26, *p* < .001, R^2^ = .26 (Adj R^2^ = .25)					

SE = Standard error.

^†^Reference = Living with family; ^‡^Reference = No.

## Data Availability

The dataset supporting the conclusions is available from the corresponding author on reasonable request.
